# Ultrahigh performance of engineered NiO and Ce_2_O_3_@NiO bimetallic nanomaterials for electrocatalytic water splitting and supercapacitor applications

**DOI:** 10.1039/d6ra01358a

**Published:** 2026-07-09

**Authors:** Kanwal Naz, Zeshan Ali Sandhu, Soha Ghaffar, Sadaf ul Nisa, Sidra Tul Muntaha, Muhammad Danish, Syed Rizwan Shafqat, Sumera Arshad, Sufyan Ashraf, Muhammad Asam Raza

**Affiliations:** a Department of Chemistry, Faculty of Science, University of Sialkot Sialkot 51310 Pakistan drdanish62@gmail.com; b Department of Chemistry, Faculty of Science, University of Gujrat, Hafiz Hayat Campus Gujrat 50700 Pakistan zeshansandhu89@gmail.com; c Jiangsu Key Laboratory of New Power Batteries, Jiangsu Collaborative Innovation Centre of Biomedical Functional Materials, School of Chemistry and Materials Science, Nanjing Normal University Nanjing 210023 China; d Interdisciplinary Research Center in Biomedical Materials (IRCBM), COMSATS University Islamabad, Lahore Campus Lahore 54000 Punjab Pakistan; e School of Photovoltaics and Renewable Energy Engineering, University of New South Wales Sydney 2052 Australia

## Abstract

A sol–gel approach was employed for the preparation of pure NiO and Ce_2_O_3_@NiO nanomaterials for multifunctional application in supercapacitors and electrocatalysis. X-ray diffraction spectroscopy (XRD) confirmed the development of bunsenite NiO. Scanning electron microscopy demonstrated a flake-like interconnected structure in the 3% Ce_2_O_3_@NiO nanomaterial, and transmission electron microscopy exhibited uniformly dispersed particles with sizes of about 30–80 nm. Electrochemical performance illustrated an excellent specific capacitance of 1403 F g^−1^ and energy density of 40.70 Wh kg^−1^ for 3% Ce_2_O_3_@NiO at a scan rate of 5 mV s^−1^. On the other hand, pure NiO and 5% Ce_2_O_3_@NiO depicted specific capacitance values of 1201 and 1290 F g^−1^, respectively. Moreover, the 3% Ce_2_O_3_@NiO electrode material achieved an overpotential of 444 mV and an onset potential of 1.44 V at 10 mA cm^−2^ for the oxygen evolution reaction (OER). Tafel slopes further elucidated the improved charge transfer efficiency of 3% Ce_2_O_3_@NiO, showing lower Tafel slope values of 112 mV dec^−1^ for the HER and 100 mV dec^−1^ for the OER, compared with those of pure NiO and 5% Ce_2_O_3_@NiO. These outcomes demonstrated the potential impact of cerium doping in NiO for electrocatalysis and energy storage systems.

## Abstract 2

A sol–gel approach was employed for the preparation of nanomaterials with multifunctional applications. A higher specific capacitance of 1403 F g^−1^ and energy density of 40.70 Wh kg^−1^ were observed for 3% Ce_2_O_3_@NiO at 5 mV s^−1^.

## Graphical abstract

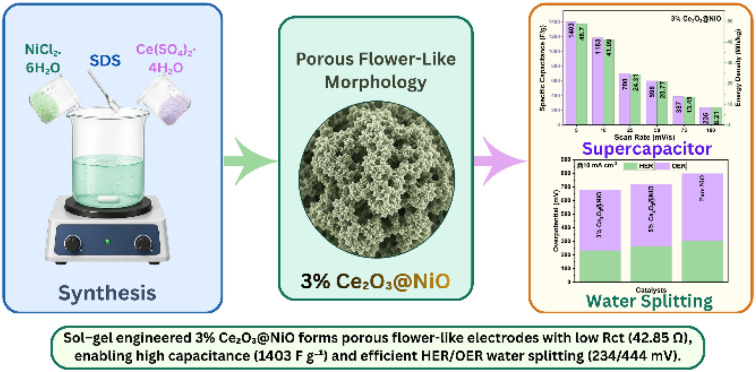

## Introduction

The depletion of fossils fuels and intensifying global climate changes are shifting the world energy preference towards renewable energy resources.^1^ A published report has show an average increase in global temperature of about 1 °C due to climate changes, and its further surge cannot be compensated without valuable preventive measures and sustainable solutions.^2^ A green and efficient approach is a dire need of this globe. In this scenario, hydrogen is considered an eco-friendly and efficient energy carrier for sustainable energy solutions.^3^ Electrochemical water splitting is a significant process employed for the generation of hydrogen fuel.^4^ The dependence on renewable energy is considered a solution for meeting today's energy demand.^5^ It ensures various advantages, like reducing the emission of greenhouse gases and enabling remote areas to access energy facilities.^6^ Various technologies have been assessed for renewable energy generation, like those based on wind and solar energy and electrolysis of water. Among these technologies, water electrolysis has been considered as the most reliable and better process for the production of hydrogen.^7^ Conversely, supercapacitors are also attractive candidates for energy solutions due to their high energy and power density, long life cycle and excellent stability and capacity retention.^8–10^ Among supercapacitors, pseudocapacitors and double layer supercapacitors show exceptional properties due to their faradaic reversible redox reaction mechanism.^11^

The electrolysis of water is really a sustainable process; the development of effective and economical electrocatalysts for water electrolysis is the current focus of researchers.^12^ Electrochemical water splitting involves two half reactions, the oxygen evolution reaction (OER) performed at the anode and the hydrogen evolution reaction (HER) performed at cathode, for the generation of H_2_.^13,14^ The OER is a critical process that requires multiple electron and proton transfer steps for hydrogen generation through the hydrogen evolution reaction. An electrocatalyst with a lower overpotential and closer value to Nernst potential may be considered superior for the electrochemical water splitting mechanism.^15^ Hybrid materials have drawn valuable interests in electrocatalysis due to their synergistic effects and unique properties like remarkable chemical stability, mechanical strength, and electronic and electrochemical performance.^16^ On the other hand, energy storage systems are performing critical roles in energy economy. Among all energy storage devices, supercapacitors are considered efficient electrode materials for electrochemical excellence.^17^

The remarkable properties of supercapacitors like exceptional power density, fast charge–discharge mechanism, higher charge retention and wide range of temperature performance have drawn the interest of researchers in energy storage and conversion devices.^18^ Supercapacitors have been categorized into two types on the basis of their energy storage mechanisms and processes: (1) electrical double-layer capacitors store charge at the electrode–electrolyte interface through electrostatic attractions, and (2) pseudocapacitors store charge *via* fast reversible processes at the electrode–electrolyte interface.^19^ Transition metal oxides (TMOs) have been considered effective electrode materials for electrochemical supercapacitor performance due to their better capacitance values and robust electrical conductivity.^20^ Conversely, hydrogen production is mainly depending on IrO_2_ (ref. 21 and 22) and RuO_2_ (ref. 23 and 24) that show superior electrocatalytic activity. Due to their high cost and fouling effect, these electrocatalysts have been replaced with other hybrid bi-metallic catalysts. In this regard, the current research has focused on the development of a cheap, accessible, long-life and non-precious electrocatalyst.^25^

Researchers have been putting in remarkable efforts to improve the electrochemical excellence of electrode materials *via* the coupling or synergistic interactions of TMOs with other carbon-based materials, conducting polymers and rare-earth metals.^26^ Moreover, NiO nanomaterials have drawn valuable interest due to their superior thermal stability, chemical stability, exceptional specific capacitance, eco-friendliness and simple synthesis. The literature survey attributes the average performance of NiO as an electrode material and electrocatalyst to its lower electrical conductivity. The performance of NiO can be further increased by the incorporation of dopants into its matrix.^27^ Recently, NiO has been modified with other electrode materials, like Ru-NiO,^28^ CNT/NiO,^29^ Fe@NiO,^30^ Cu@NiO^31^ and Ce-doped NiO,^32^ for assessing its electrochemical and electrocatalytic performance. The conjugation of other materials induced structural changes in the matrix of NiO, with improved surface area and active sites resulting in its increased performance.^33^

Various synthetic methods have been employed for the synthesis of TMOs, such as the hydrothermal,^34^ micro emulsion,^35,36^ chemical vapour deposition,^37^ solvothermal^38,39^ precipitation^40^ and sol–gel^41^ methods. Among these synthetic approaches, sol–gel methodology is more accessible and reliable because it tailors the crystallinity, shape, size and dopant concentration of the prepared nanomaterials.^42^ The current research tailored the synthesis of pure and hybrid nanomaterials for multifunctional applications. The prepared Ce-enriched NiO nanomaterials incorporated extra actives sites and improved surface area that induced superior supercapacitor and water splitting performance compared with that of previously reported electrodes. The research aims to assess the multifunctionality of the prepared electrodes in both supercapacitor and water splitting applications *via* assessing their electrochemical performance, electrocatalytic efficiency and charge storage ability. This research overcomes the challenges faced by NiO as a multifunctional electrode material.

## Experimental details

### Materials and methods

Pure nickel oxide (NiO) and cerium-enriched nickel oxide (Ce_2_O_3_@NiO) were synthesized using various metal precursor salts. The metal precursor salts used include nickel chloride hexahydrate (NiCl_2_·6H_2_O) and cerium sulphate tetrahydrate (Ce(SO_4_)_2_·4H_2_O) with ≥99% purity. All the precursors were purchased from Sigma-Aldrich. Sodium dodecyl sulphate (NaC_12_H_25_SO_4_) and ethanol (C_2_H_5_OH) were used as the surfactant and solvent, respectively. The reagents and precursor salts were used without any modification or alterations.

### Synthesis of NiO and Ce_2_O_3_@NiO bimetallic nanomaterials

#### Synthesis of the pure NiO nanomaterial

**Fig. 1:**
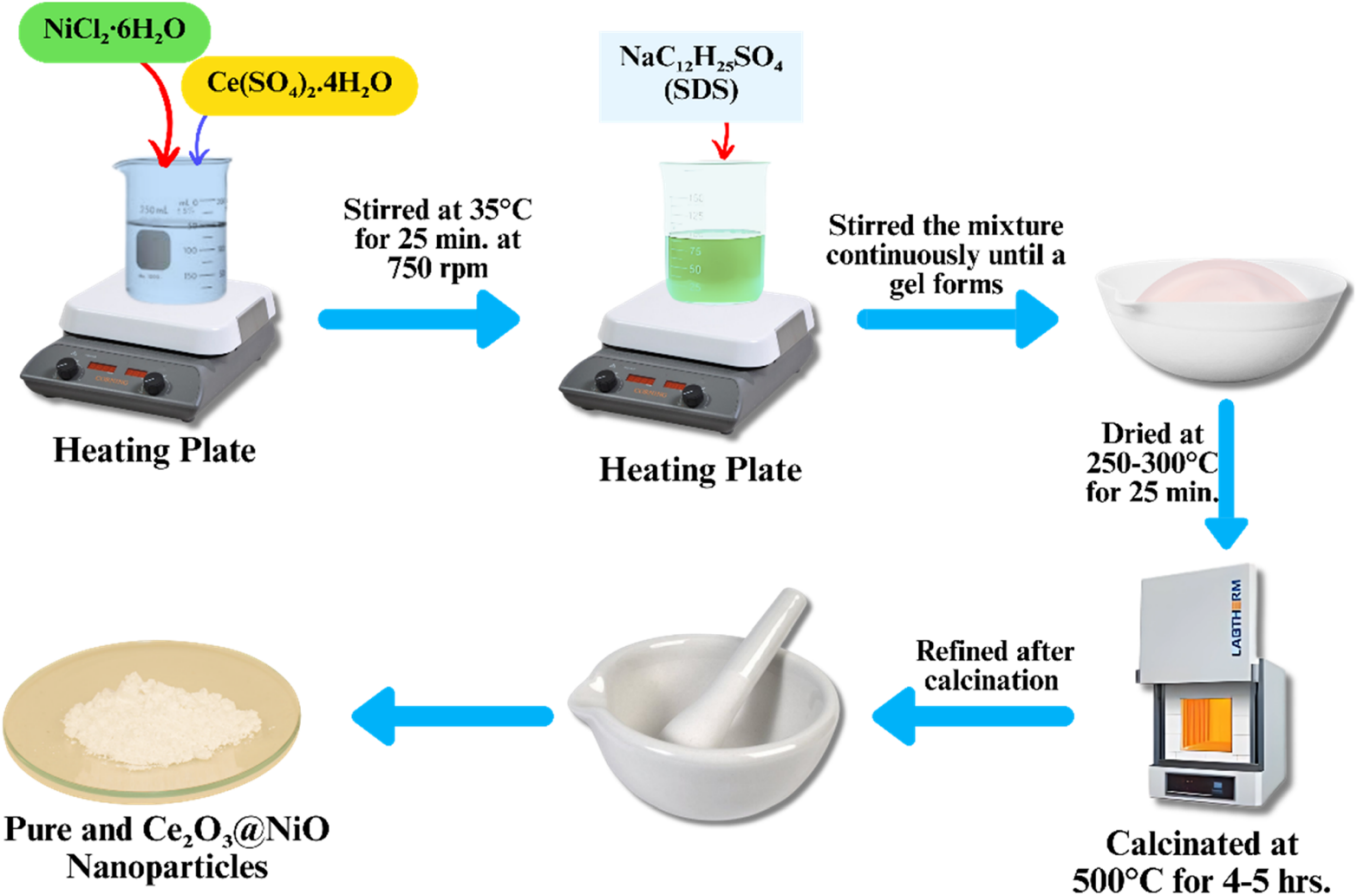
Schematic of the sol–gel approach for the synthesis of the prepared nanomaterials.

The pure NiO nanomaterial was prepared *via* a modified sol–gel approach.^18^ A 0.6 M optimized solution of nickel chloride hexahydrate was prepared in deionized water. The solution was placed on a heating plate under continuous stirring at 750 rpm for 25 min at 35 °C. After the homogenization of this solution, a specific amount of sodium dodecyl sulphate was added in pinches with continuous stirring. SDS worked as the surfactant and facilitated gel formation. The prepared gel was transferred into a china dish for heating at 250–300 °C for 25–30 min. The dried gel was then crushed into a fine powder for calcination at 500 °C for 4–5 hours. The calcination removed chlorides, sulphates and other impurities from the material. Finally, the calcined product was again crushed into a fine powder, and the prepared nanomaterial was characterized to confirm its morphology and structure. The schematic of the sol–gel methodology is depicted in Fig. 1.

#### Synthesis of the Ce_2_O_3_@NiO bimetallic nanomaterials

Ce_2_O_3_@NiO was prepared using the same approach at varying concentration of a precursor salt. For the preparation of the cerium-enriched NiO nanomaterials, a 0.6 M solution of nickel chloride hexahydrate (NiCl_2_·6H_2_O) and cerium sulphate tetrahydrate (Ce(SO_4_)_2_·4H_2_O) was collectively prepared in deionized water. The solution of the bi-metallic precursor salt was later placed on the heating plate for homogenization *via* stirring. The subsequent procedures were similar to those followed in the synthesis of the pure NiO nanomaterial.

### Preparation of the working electrode on a nickel foam

**Fig. 2:**
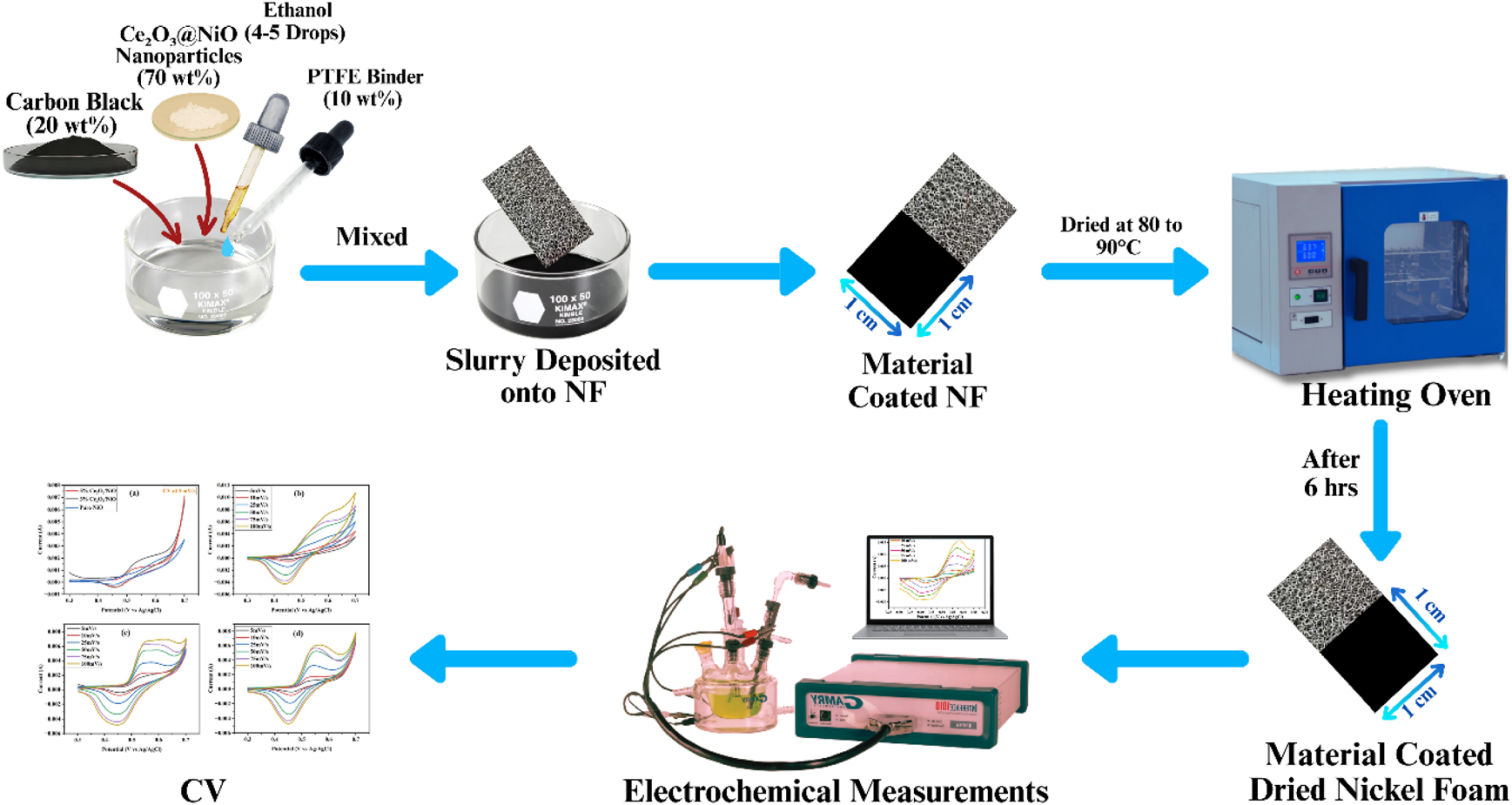
Representation of the working electrode preparation strategy using nanomaterials, carbon black and the PTFE binder.

The electrochemical performance was assessed *via* preparing the working electrode material on a nickel foam. The pure and doped working electrodes were prepared *via* slurry formation, incorporating the nanomaterials (0.003 g) 75%, carbon black (15%) and polytetrafluoroethylene binder (PTFE 10%), respectively. The prepared slurry was used as a standard for the assessment of electrochemical performance. A standard ratio of PTFE 10% was used to enable binding strength without hindering the active sites on the electrode materials. The materials with specific ratio were mixed and pasted on NF in a 1 × 1 cm^2^ dimension. The pasted electrode was later dried at 80–100 °C for 10 h. After drying, the electrode materials underwent CV, EIS and LSV analyses. Fig. 2 illustrates the schematic of the working electrode preparation.

## Results and discussion

**Fig. 3:**
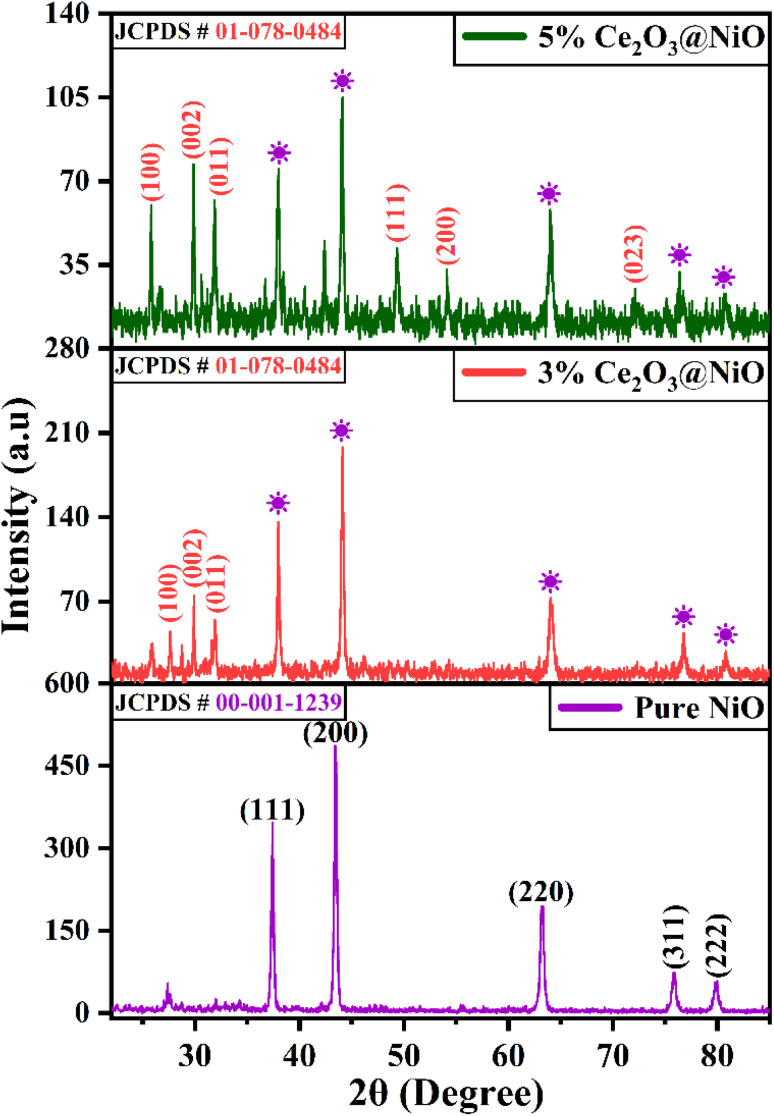
XRD diffraction patterns of the synthesized NiO and Ce_2_O_3_@NiO (3 and 5%) nanomaterials.

XRD analysis was carried out to investigate the structural features of pure NiO, 3% Ce_2_O_3_@NiO, and 5% Ce_2_O_3_@NiO, as shown in Fig. 3. Pure NiO shows distinct diffraction peaks at 37.44°, 43.47°, 63.2°, 75.37°, and 79.87°, corresponding to the (111), (200), (220), (311), and (222) planes of the cubic bunsenite NiO, which are in good agreement with JCPDS no. 00-001-1239.^43^ For the cerium-based samples, the NiO reflections are retained, confirming that the pure NiO structure was maintained after the incorporation of cerium. There are only some weak additional reflections are indexed for the doped materials, namely (100), (002), (011), (111), and (200) planes (JCPD no. 01-078-0484).^44^ A notable feature of the doped nanomaterials is the reduction in diffraction peak intensities relative to pure NiO, which suggests lowered crystallinity and increased structural disorder. This reduction may arise from dopant-induced defects, lattice distortion, and microstrain, which hinder long-range crystalline ordering. At higher cerium concentrations, this effect is more pronounced: 5% Ce_2_O_3_@NiO shows a higher peak intensity than 3% Ce_2_O_3_@NiO, suggesting a stronger disruption in the NiO lattice. Within the cerium-containing compositions, the 3% Ce_2_O_3_@NiO material seems to offer a more balanced structure than 5% Ce_2_O_3_@NiO. The relatively small decline in diffraction intensity indicates that cerium incorporation at a lower level (3% Ce_2_O_3_@NiO) creates advantageous defect sites without significantly disrupting the NiO crystal structure, while a higher amount of cerium doping (5% Ce_2_O_3_@NiO) causes increased structural disorder responsible for the amorphous-like behaviour. This retained crystallinity along with moderate defect generation also correlates with the higher electrochemical and electrocatalytic response of the 3% Ce_2_O_3_@NiO nanomaterial than that of the 5% Ce_2_O_3_@NiO nanomaterial.^45^

**Fig. 4:**
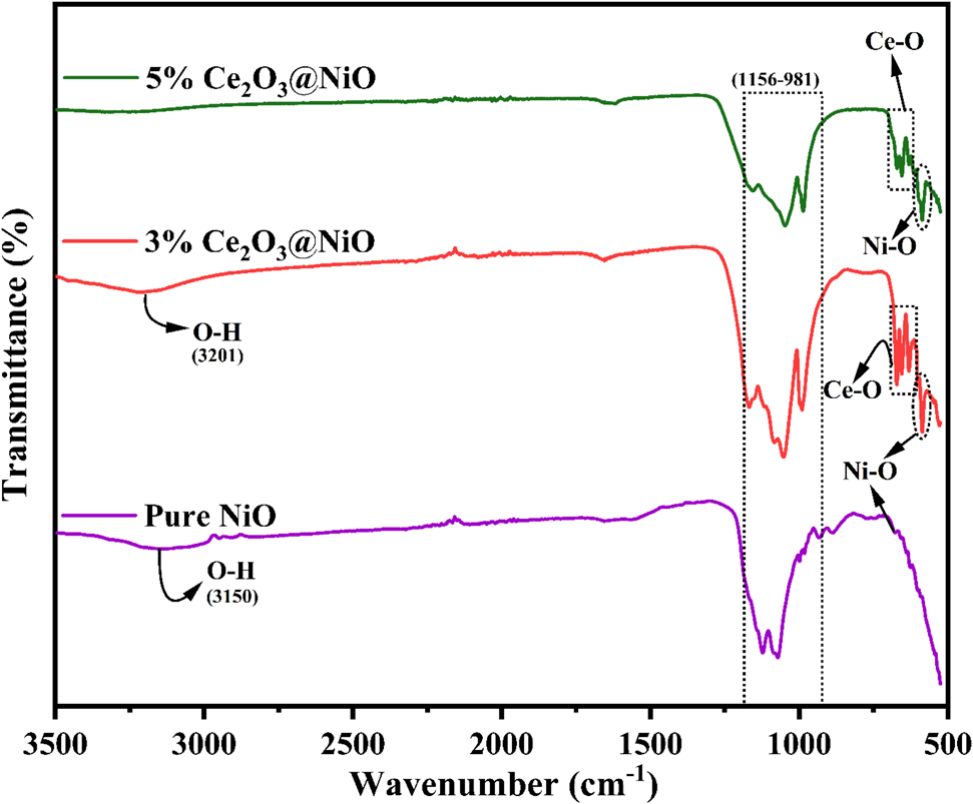
FTIR spectrum of the synthesized NiO and Ce_2_O_3_@NiO (3% and 5%) nanoparticles.

FTIR spectroscopy was performed to investigate the presence of functional groups present in the prepared nanomaterials. Fig. 4 depicts the FTIR analysis of the pure NiO, 3% Ce_2_O_3_@NiO and 5% Ce_2_O_3_@NiO nanomaterials scanned in the range of 3500 to 500 cm^−1^.^46^ The broad bands observed at 3150 and 3201 cm^−1^ are associated with the O–H stretching vibrations, indicating the presence of water molecules in pure NiO and 3% Ce_2_O_3_@NiO. The weak bands observed in the region of 1156–981 cm^−1^ correspond to the residual surface species or adsorbed atmospheric species, including possible carbonate/bicarbonate-type C–O vibrations, as commonly reported for ceria-containing oxide nanomaterials.^47^ The peak at 682 cm^−1^ in the pure NiO spectra and prominent bands at 583 cm^−1^ in both 3% and 5% Ce_2_O_3_@NiO are assigned to Ni–O stretching vibration modes, providing strong evidences regarding the presence of the NiO nanocrystalline phase.^45,48^ The characteristic absorption bands of Ce_2_O_3_ observed at 674 and 626 cm^−1^ in 3% and 5% Ce_2_O_3_@NiO suggest the presence of cerium–oxygen bonding.^49^

**Fig. 5:**
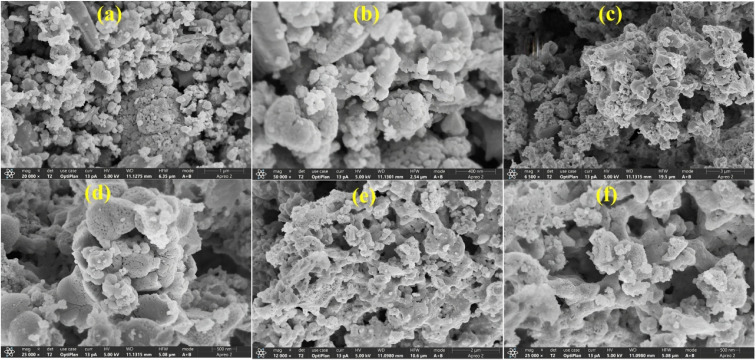
SEM analysis of the pure and doped nanomaterials: (a and b) pure NiO, (c and d) 3% Ce_2_O_3_@NiO, and (e and f) 5% Ce_2_O_3_@NiO.

Scanning electron microscopy (SEM) was employed for the morphological assessment of the pure and doped nanomaterials. Fig. 5(a and b) depict the irregular agglomerated shape morphology of the pure NiO nanomaterial. The pure NiO nanomaterial surface appears rough and granular, indicating monocrystalline domain formation. The SEM image of 3% Ce_2_O_3_@NiO illustrated in Fig. 5(c and d) showed a remarkable transformation in morphology. A uniform distribution of particles was observed with a porous, loosely packed, and flower-like interconnected structure. The flower-like morphology in 3% Ce_2_O_3_@NiO improves its electrochemical excellence, recommending it as a more reliable material for water splitting and supercapacitor applications. Conversely, the SEM analysis of 5% Ce_2_O_3_@NiO is depicted in Fig. 5(e and f). The increase in cerium doping in NiO resulted in a collapsed and agglomerated structure of the material. The larger spongy like network structure exhibited in the material with higher cerium doping may induce barrier in the transportation of charges and ion diffusion. Thus, the 3% Ce_2_O_3_@NiO nanomaterial depicted a more refined and uniform morphology, playing a vital role in its superior electrochemical and water splitting performance.

**Fig. 6:**
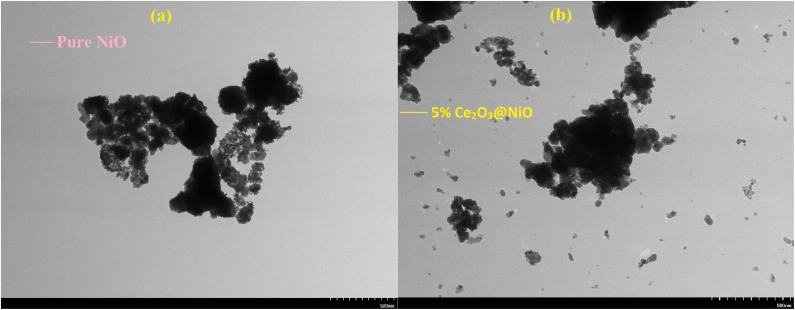
TEM analysis of the pure and doped nanomaterials: (a) pure NiO and (b) 3% Ce_2_O_3_@NiO at 500 nm.

Transmission electron microscopy (TEM) was performed to define the morphological variations in the pure and doped nanomaterials. Fig. 6(a) reveals the TEM analysis of pure NiO, demonstrating dense agglomeration in the nanomaterial with relatively large particle size (50–100 nm) and irregular clusters. The rigid aggregation of particles lowers the surface area, which may have restricted electrochemical and water splitting excellence. Conversely, 3% cerium-doped NiO illustrated in Fig. 6(b) demonstrated more uniform and dispersed structures with smaller particle sizes of about 30–80 nm. The doping of cerium in NiO may lower the agglomeration of particles, improve surface active sites, and enhance the penetration of the electrolyte. The improvement in structure induced significant enhancements in catalytic and charge transfer performance; thus, the 3% cerium-doped NiO nanomaterial is more reliable and excellent for multifunctional applications.

### Electrochemical performance of the NiO and Ce_2_O_3_@NiO bimetallic nanomaterials

**Fig. 7:**
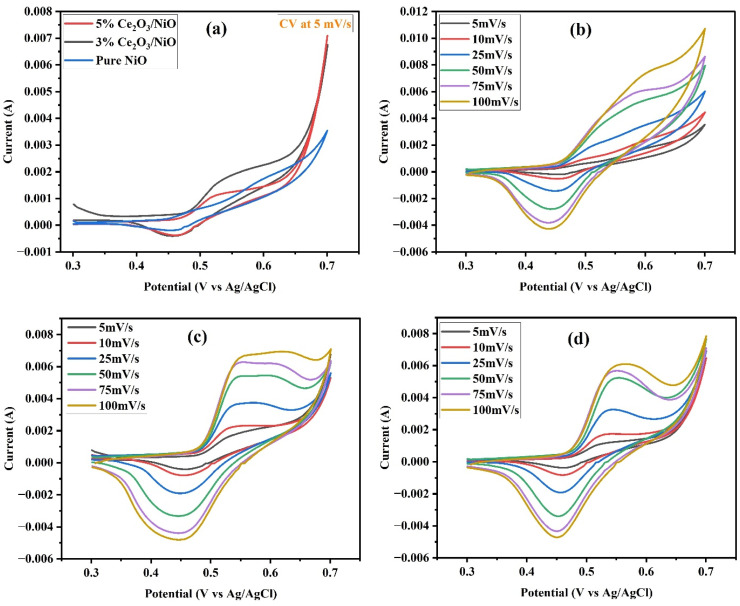
CV curves of the prepared nanomaterials in 2 M KOH: (a) comparative CV curves of pure NiO, 3% Ce_2_O_3_@NiO and 5% Ce_2_O_3_@NiO at 5 mV s^−1^, (b) CV curves of pure NiO, (c) CV curves of 3% Ce_2_O_3_@NiO, and (d) CV curves of 5% Ce_2_O_3_@NiO.

The electrochemical excellence of the prepared working electrodes on the nickel foam was observed using cyclic voltammetry. The electrochemical performance was evaluated using a three electrode system consisting of a working electrode (nickel foam), a reference electrode (Ag/AgCl) and a counter electrode (Pt wire). The CV curves were assessed at different scan rates of 5–100 mV s^−1^ in an electrolytic solution of 2 M KOH within a potential window of 0.3–0.7 V. The working electrode was prepared using carbon black, nanomaterial and polytetrafluoroethylene binder in a specific ratio. A 3 mg cm^−2^ of nanomaterial was deposited on the nickel foam for assessing the electrochemical performance without IR compensation. The OH^−^ ions present in the electrolyte solution play a pivotal role in facilitating the redox reactions, particularly in metal oxide nanomaterials. It improves the electron kinetic mechanism due to enhanced conductivity that ensures the symmetrical shapes of CV curves. The pure nickel oxide electrode material may exhibit a battery-type faradaic behaviour, but the effect of doping or composite formation introduced extra vacancies in the material that directly relates to a pseudocapacitive behaviour rather than the battery-type behaviour. In the present study, the electrode materials demonstrated symmetrical shapes of CV at varying scan rates, indicating the pseudocapacitive behaviour of the materials. The higher pH of the electrolyte solution stabilizes the electrodes and induces a change in the redox potential, directly improving the pseudocapacitive and catalytic performance of the prepared nanomaterials.^40^ The appearance of multiple redox peaks indicates complex faradaic processes, and in pure NiO, a single pair of redox peaks exhibited a reversible transformation in nickel oxide between Ni^2+^/Ni^3+^ and Ni^3+^/Ni^2+^.^50,51^ Similarly, Ce_2_O_3_@NiO demonstrated multiple broad peaks owing to the incorporation of cerium into its matrix. The presence of the redox pairs of cerium Ce^3+/^Ce^4+^ results in multiple redox couples overlapping with nickel. In this regard, oxygen vacancies are produced due to lattice strain induced by doping that directly facilitate charge storage capacity and electrochemical performance. Fig. 7 depicts the CV curves of the pure and doped nanomaterials.

The 3% Ce_2_O_3_@NiO nanomaterial showed higher *I*–*V* responses than pure NiO and 5% Ce_2_O_3_@NiO, confirming its better specific capacitance and energy density values. The increase in specific capacitance involves improved active sites and surface area that directly decrease the defects in doped nanomaterials. The specific capacitance of the prepared electrode materials was calculated with the reported eqn (1).^52^1
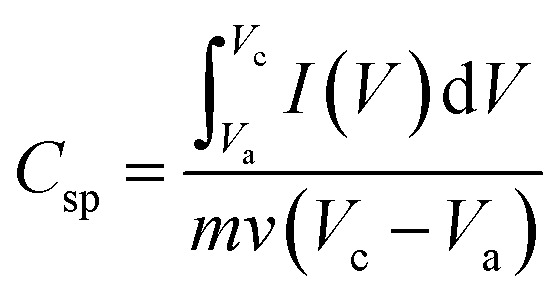
where *m* represents the active mass of the electrode material, *v* depicts the scan rate and *V*_c_ − *V*_a_ illustrates the potential window of the electrochemical performance.

Pure NiO, 3% Ce_2_O_3_@NiO and 5% Ce_2_O_3_@NiO exhibited remarkable specific capacitance values of 1201 F g^−1^, 1403 F g^−1^ and 1290 F g^−1^, respectively. The 3% Ce_2_O_3_@NiO electrode material demonstrated increased capacitance than pure NiO and 5% Ce_2_O_3_@NiO. The doping of cerium into the nickel matrix introduced new active sites and increased the surface area that led to enhancement in specific capacitance values. The higher doping of 5% Ce_2_O_3_@NiO may offer another phase in the metal matrix that decreases the number of active sites and reduces surface area, thereby reducing its specific capacitance. The variation in the CV curves with varying specific capacitance was observed along a distinct scan rate. The increase in scan rate from 5–100 mV s^−1^ exhibited a shift in the oxidation peaks towards higher potential and reduction peaks towards lower potential. The major distinction between the oxidation and reduction potential of peaks demonstrated the irreversibility of the redox reaction. The decrease in *C*_sp_ at higher scan rates indicated the instability of internal active sites, which leads to the successful redox reaction. The electrode surface plays a critical role in the charge–discharge process during the redox reaction at higher scan rates.^36^ The energy density of the prepared electrode can be measured from specific capacitance using the reported eqn (2).^53,54^2
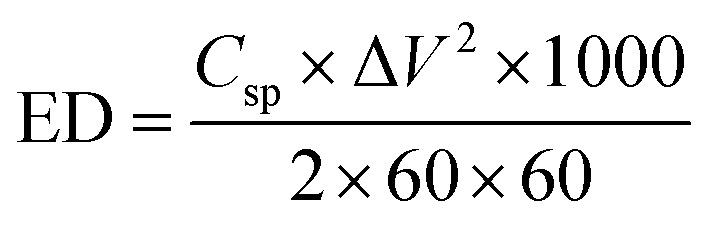


**Fig. 8:**
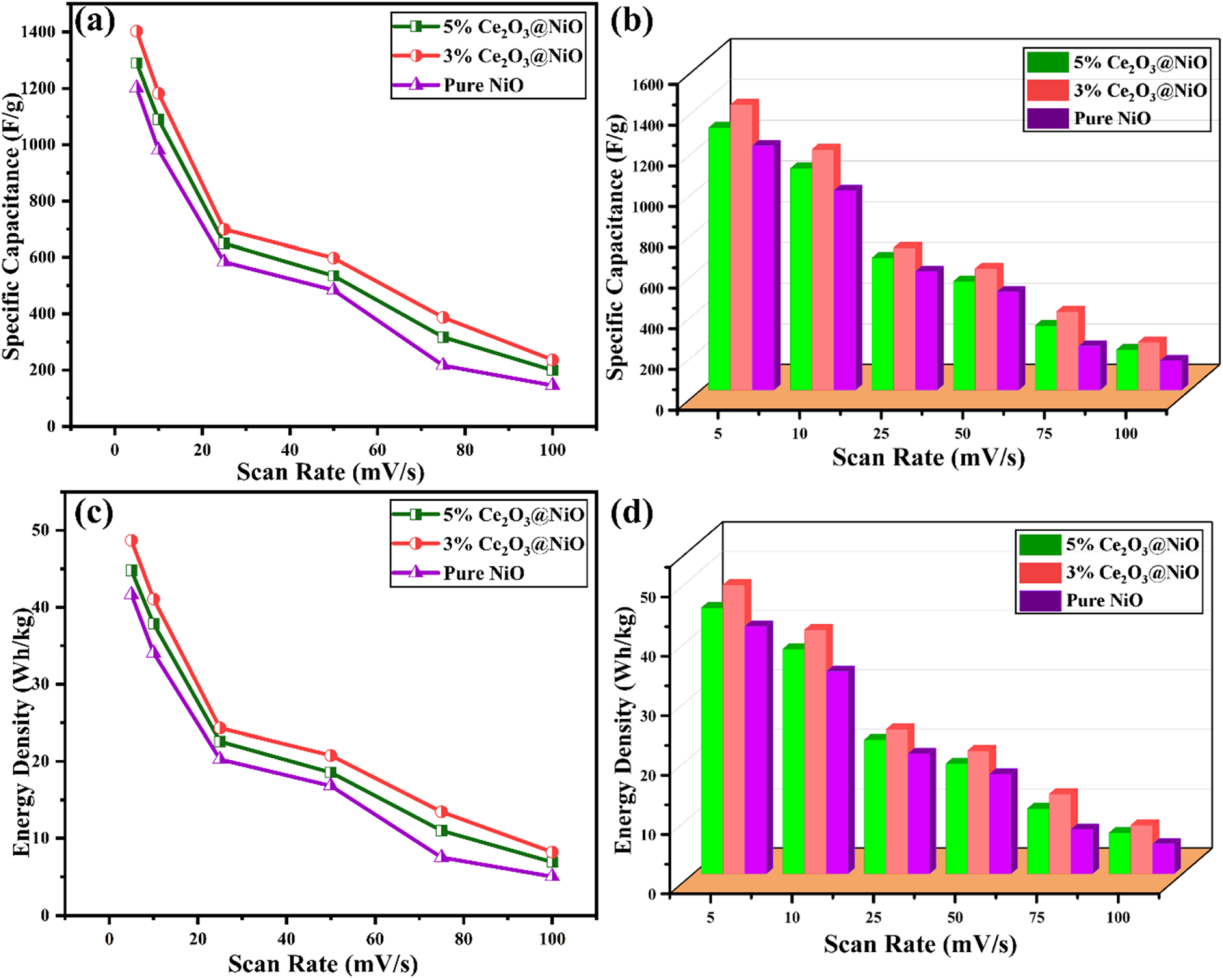
Comparative study of capacitance and energy density at different scan rates: (a and b) scan rate *vs.* specific capacitance plots and (c and d) scan rate *vs.* energy density plots.

Moreover, the trend in energy density is associated with specific capacitance. The increase in scan rate directly declines the energy density value of electrodes. At higher scan rates, the ions have limited time to accumulate at the active sites on the electrode surface, resulting in restricted capacitance and energy density values.^55^Fig. 8 illustrates the scan rate *vs.* energy density and specific capacitance plots. A comparative analysis is performed between the electrochemical performance of 3% Ce_2_O_3_@NiO and previous benchmark Ni-based nanomaterials in Table 1.

**Table 1:** Comparative study of 3% Ce_2_O_3_@NiO developed in the present research with documented Ni-based nanomaterials for electrochemical performance analysis

Nanomaterial	Methodology	Electrolyte	Capacitance (F g^−1^)	Reference
CeO_2_@NiO	Hydrothermal	1 M KOH	317	56
Ni-doped CuO	Chemical precipitation	2 M KOH	396.67	57
Ti_2_O_3_–CeO_2_	Hydrothermal	—	517	58
Mn-doped NiO	Chemical precipitation	2 M Na_2_SO_4_	555	59
Bi_2_O_3_@Ce_2_O_3_	Precipitation	2 M KOH	915	60
CeO_2_–MnO_2_-NG	Hydrothermal	—	772	61
La_2_O_3_–CeO_2_	Microwave irradiation	3 M KOH	1005	62
5% CeO_2_-doped Co_3_O_4_	Sol–gel	1 M KOH	1097	63
Fe-doped NiO NPs	Precipitation	3 M KOH	1200	64
RGO/β-Ni(OH)_2_/CeO_2_	Hydrothermal	3 M KOH	1250	65
3% Ce_2_O_3_@NiO	Sol–gel	2 M KOH	1403	This work

**Fig. 9:**
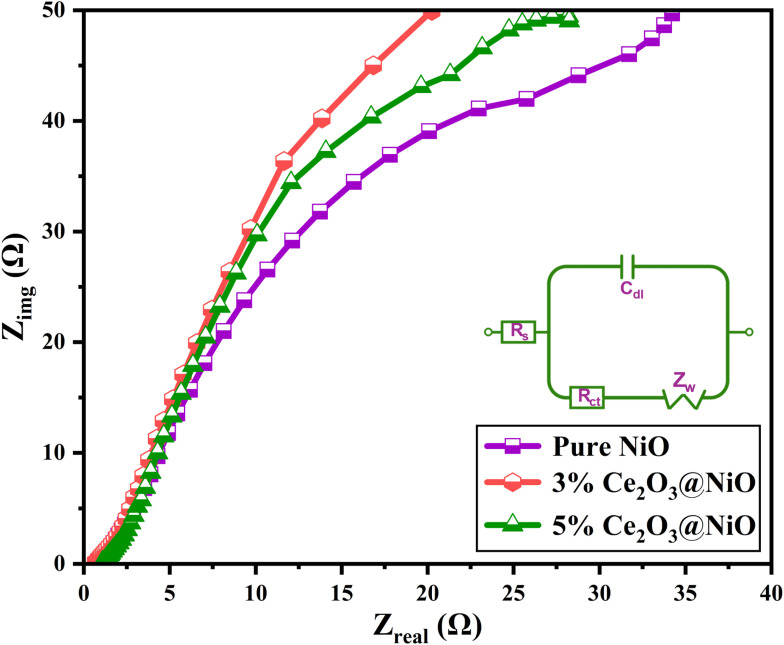
Electrochemical impedance spectroscopy analysis and circuit diagram of the prepared nanomaterials.

Electrochemical impedance spectroscopy (EIS) was used to determine the charge kinetic mechanism, diffusion of ions and internal electrode–electrolyte resistance in the electrochemical system (Fig. 9). The Nyquist plots of the pristine NiO, 3% Ce_2_O_3_@NiO and 5% Ce_2_O_3_@NiO were fitted using the *R*_s_–(*R*_ct_‖CPE)–*W* equivalent circuit model, where *R*_s_ is the solution resistance, *R*_ct_ is the charge-transfer resistance, CPE represents the constant phase element accounting for non-ideal interfacial capacitive behaviour and *W* stands for Warburg diffusion element. The fitted *R*_s_ of pure NiO, 3% Ce_2_O_3_@NiO and 5% Ce_2_O_3_@NiO are 1.0890, 0.83991 and 0.9776 Ω, respectively, and the *R*_ct_ values for the same compounds are 52.81, 42.85 and 49.81 Ω, respectively. The 3% Ce_2_O_3_@NiO electrode had the lowest *R*_s_ and *R*_ct_ values among all samples, suggesting a reduced resistance between its electrolyte/electrode surface and favourable interfacial charge-transfer kinetics. The lower *R*_ct_ of 3% Ce_2_O_3_@NiO confirms that there is a faster flow of electrons at the electrode/electrolyte interface, which is beneficial to both pseudocapacitive charge storage and electrocatalytic water-splitting reactions. In addition, the Warburg region in the low-frequency domain reflects ion-diffusion behaviour, and the comparatively improved impedance response of 3% Ce_2_O_3_@NiO suggests its more efficient ion transport than pure NiO and the 5% Ce_2_O_3_@NiO. These results demonstrate that moderate cerium incorporation optimizes the electrochemical interface by lowering the internal and charge-transfer resistances without introducing excessive structural disorder. Consequently, the 3% Ce_2_O_3_@NiO sample exhibits the most favourable impedance characteristics, which is consistent with its excellent supercapacitor and electrocatalytic performance.

### Water splitting performance of the pure NiO and Ce_2_O_3_@NiO bimetallic nanomaterials

#### Oxygen evolution reaction

**Fig. 10:**
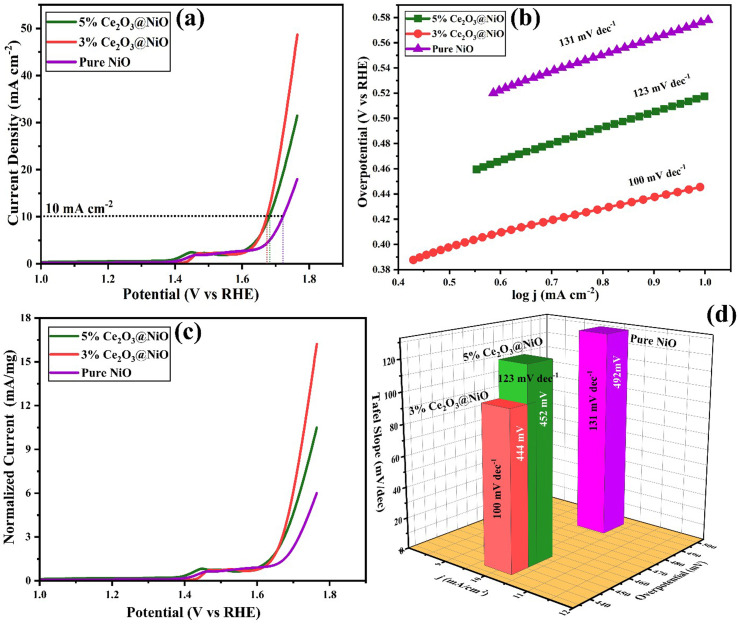
(a) LSV polarization curves of pure NiO, Ce_2_O_3_@NiO (3% and 5%) for the OER, (b) corresponding Tafel plots of pure NiO and Ce_2_O_3_@NiO (3% and 5%) using 2 M KOH at 5 mV s^−1^ scan rate, (c) normalized current curves drawn against potential values of pure NiO and Ce_2_O_3_@NiO (3% and 5%) and (d) comparison bar graph of the overpotential and Tafel slope values of pure NiO and Ce_2_O_3_@NiO (3 and 5%).

The linear sweep voltammetry (LSV) analysis of pure NiO, 3% Ce_2_O_3_@NiO, and 5% Ce_2_O_3_@NiO was observed in a 2.0 M KOH electrolyte solution at a scan rate of 5 mV s^−1^, as shown in Fig. 10(a). It is evident that the pure NiO-modified electrode exhibits lower electrocatalytic activity and requires a high onset potential of 1.46 V *vs.* RHE and an overpotential of 492 mV at 10 mA cm^−2^ for water oxidation. Alternatively, the 3% and 5% Ce_2_O_3_@NiO-modified electrodes display lower onset potentials of 1.44 V and 1.45 V *vs.* RHE, with corresponding overpotential of 444 mV and 452 mV, respectively. Particularly, the 3% Ce_2_O_3_@NiO electrocatalyst requires only 444 mV overpotential to facilitate the OER at 10 mA cm^−2^, demonstrating significant oxygen evolution performance compared to pure NiO and 5% Ce_2_O_3_@NiO.^2,66^ This improvement is linked with improved electrical conductivity and the presence of abundant active sites. Moreover, the normalized current curves drawn against potential values confirm that 3% Ce_2_O_3_@NiO exhibits a higher catalytic activity compared to other electrocatalysts based on mass activity, as depicted in the Fig. 10(c). Consequently, the 3% Ce_2_O_3_@NiO electrocatalyst shows a better electrocatalytic activity over pure NiO and 5% Ce_2_O_3_@NiO. Tafel plots were derived from polarization curves through the Tafel equation (*η* = *b* × log *j* + *a*), where *j* denotes the current density, *b* represents the Tafel slope, and *η* is the overpotential, to understand the reaction kinetics of the prepared electrocatalysts. The Tafel plots reveal that 3% Ce_2_O_3_@NiO exhibits a lower Tafel slope of 100 mV dec^−1^ compared to 123 mV dec^−1^ for pure NiO and 131 mV dec^−1^ for 5% Ce_2_O_3_@NiO, as illustrated in Fig. 10(b). The reaction kinetics of 3% Ce_2_O_3_@NiO electrocatalyst demonstrate that it is more favourable than the other catalysts, exhibiting faster electron transfer and enhanced catalytic efficiency.^67^ Shanshan *et al.*, (2025) used a hydrothermal approach for the synthesis of NiO, Ce-NiO-1, Ce-NiO-2, and Ce-NiO-3 nanomaterials. The prepared materials were investigated for their electrochemical performance, particularly in the oxygen evolution reaction. The Ce-NiO-3 nanomaterial demonstrated an overpotential of 348.6 mV at 200 mA cm^−2^.^68^ The mechanism of the OER in alkaline media is a multi-step electron transfer process, which involves the oxidation of hydroxide ions to molecular oxygen. The presence of Ce_2_O_3_ is crucial for the OER activity because it facilitates the oxidation of Ni^2+^ to Ni^3+^. The reaction mechanism is given as follows:

Adsorption of OH^−^ ions

3 Ni^3+^ + OH^−^ → Ni–OH + e^−^

Formation of oxyhydroxide intermediate:

4 Ni–OH + OH^−^ → NiOOH + e^−^

Oxygen evolution through oxygenated intermediates

5 2NiOOH → 2Ni^3+^ + O_2_ + 2e^−^

Regeneration of active sites

6 Ni^3+^ + OH^−^ → Ni–OH

The interaction between Ce_2_O_3_ and NiO significantly improves the catalytic efficiency of the OER process due to enhanced charge transfer and stabilizes the active NiOOH species.^66,69^

#### Hydrogen evolution reaction

**Fig. 11:**
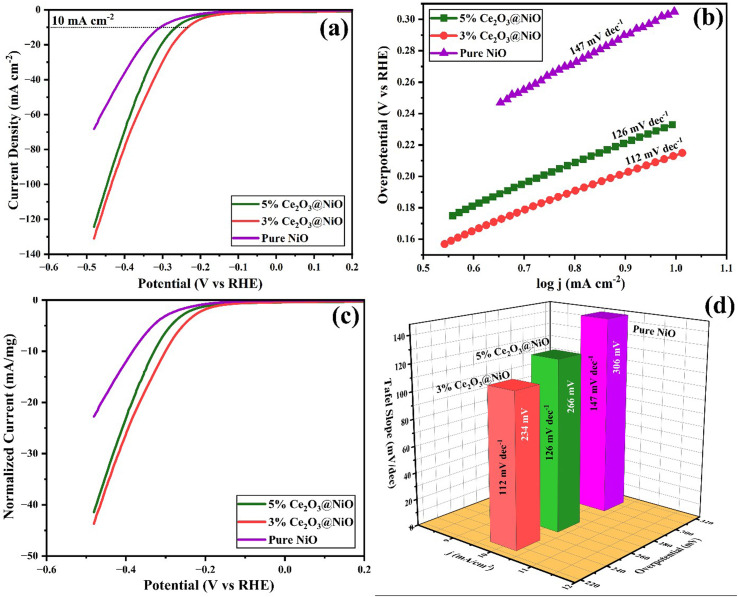
(a) LSV polarization curves of pure NiO and Ce_2_O_3_@NiO (3% and 5%) for the HER, (b) corresponding Tafel plots of pure NiO and Ce_2_O_3_@NiO (3% and 5%) using 2 M KOH at 5 mV s^−1^ scan rate, (c) normalized current curves drawn against potential values of pure NiO and Ce_2_O_3_@NiO (3% and 5%) and (d) comparison bar graph of overpotential and Tafel slope values of pure NiO and Ce_2_O_3_@NiO (3% and 5%).

The electrocatalytic performance of 3% Ce_2_O_3_@NiO for the hydrogen evolution reaction (HER) was evaluated in a 2.0 M KOH solution at 5 mV s^−1^ scan rate. It is evident that 3% Ce_2_O_3_@NiO exhibits a better HER activity compared to pure NiO and 5% Ce_2_O_3_@NiO, achieving a low overpotential of 234 mV at 10 mA cm^−2^, as represented in Fig. 11(a). In contrast, pure NiO and 5% Ce_2_O_3_@NiO require overpotentials of 266 mV and 306 mV, respectively. Additionally, the mass activity curves indicate that 3% Ce_2_O_3_@NiO outperforms the other catalysts, as illustrated in Fig. 11(c) The 3% Ce_2_O_3_@NiO catalyst exhibits a decreased Tafel slope value of 112 mV dec^−1^, which proves its good activity and better charge transfer compared to NiO (126 mV dec^−1^) and 5% Ce_2_O_3_@NiO (147 mV dec^−1^), as presented in Fig. 11(b).^70,71^ In an alkaline medium, the HER occurs through the Volmer–Heyrovsky or Volmer–Tafel mechanism, where hydrogen ions are adsorbed on the catalyst surface and subsequently combined to form molecular hydrogen. Stabilized by Ce_2_O_3_@NiO, the reaction achieves better electron transfer capabilities and hydrogen adsorption through their improved electronic properties.

7 M + H_2_O + e^−^ → M–H_ads_ + OH^−^ (Volmer)

8 M–H_ads_ + H_2_O + e^−^ → M + H_2_ + OH^−^ (Heyrovsky)

9 2M–H_ads_ → 2M + H_2_ (Tafel)

Here, M is the active catalytic site that improves proton adsorption and hydrogen gas evolution. The presence of Ce_2_O_3_ in 3% Ce_2_O_3_@NiO improves electron transfer and reduces energy barriers, thereby accelerating the HER process.^69^

**Fig. 12:**
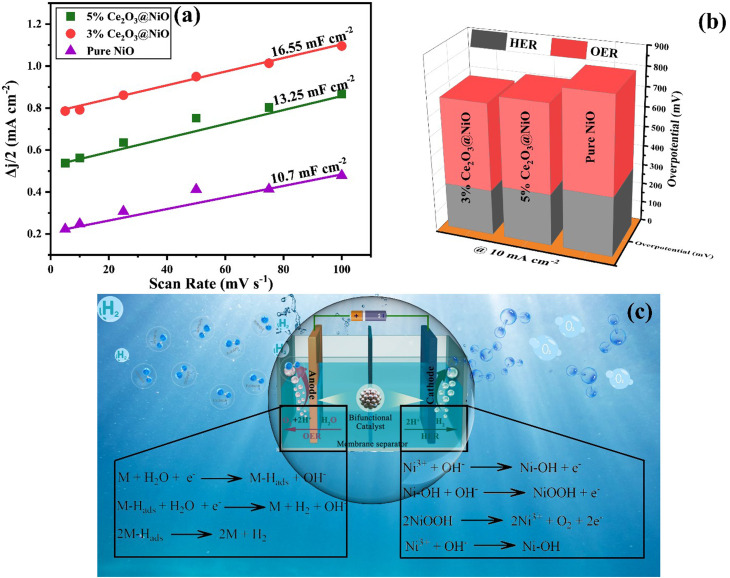
(a) *C*_dl_ curves of pure NiO and Ce_2_O_3_@NiO (3% and 5%), (b) overpotential comparison between the OER and HER of pure NiO and Ce_2_O_3_@NiO (3% and 5%), and (c) water electrolyzer operations and reaction mechanisms, with the OER depicted on the right side and the HER depicted on the left side.

To further analyze the electrochemical performance, CV tests were conducted in the non-faradaic zone at the scan rates of 5, 10, 25, 50, 75, and 100 mV s^−1^, as demonstrated in Fig. 12(a). The obtained CV curves of pure NiO, 3% Ce_2_O_3_@NiO, and 5% Ce_2_O_3_@NiO were used to determine the double-layer capacitance (*C*_dl_) values, which were 10.7 mF cm^−2^, 16.55 mF cm^−2^, and 13.25 mF cm^−2^, respectively. The higher *C*_dl_ value of 3% Ce_2_O_3_@NiO indicates a greater number of active sites, depicting its excellent electrocatalytic performance. Additionally, the electrochemical active surface area (ECSA) was calculated using eqn (10).10
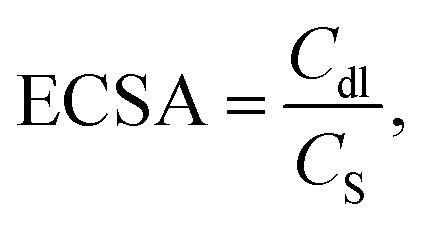
where *C*_S_ is typically 0.04 mF cm^−2^ in a 2.0 M KOH electrolyte. The estimated ECSA values of pure NiO, 3% Ce_2_O_3_@NiO, and 5% Ce_2_O_3_@NiO were 267.5 cm^2^, 413.75 cm^2^, and 331.25 cm^2^, respectively. The 3% Ce_2_O_3_@NiO electrocatalyst showed an increased ECSA value, which strengthens its catalytic performance. This catalyst shows better OER and HER behaviour due to its enhanced surface area together with its high conductivity and multiple active sites present on its surface.^70^

## Conclusion

In this study, a sol–gel methodology was employed to prepare the pure NiO and Ce_2_O_3_@NiO nanomaterials. The prepared nanomaterials were confirmed with various characterization analyses. The functional group identification was performed *via* Fourier transform infrared spectroscopy that visualized peak bands at 682 cm^−1^ and 583 cm^−1^ assigned to Ni–O vibration stretching modes, which confirmed the presence of the NiO nanocrystalline phase in the prepared materials. Cyclic voltammetry demonstrated redox reactions that involved efficient charge kinetics and stability. The pure NiO, 3% Ce_2_O_3_@NiO and 5% Ce_2_O_3_@NiO nanomaterials showed specific capacitance of 1201, 1403, and 1290 F g^−1^, respectively. On the other hand, the electrocatalytic performance of the electrode materials was assessed by evaluating their OER and HER performances. The 3% Ce_2_O_3_@NiO electrocatalyst exhibited an overpotential value of 444 mV to facilitate the OER at 10 mA cm^−2^. Similarly, the CV curves of pure NiO, 3% Ce_2_O_3_@NiO, and 5% Ce_2_O_3_@NiO were employed to calculate the double-layer capacitance (*C*_dl_), which was 10.7 mF cm^−2^, 16.55 mF cm^−2^, and 13.25 mF cm^−2^, respectively. The excellent *C*_dl_ value of 3% Ce_2_O_3_@NiO indicates its remarkable electrocatalytic performance.

## Author contributions

Kanwal Naz contributed to methodology, synthesis and data curation. Zeshan Ali Sandhu wrote the main manuscript and performed data curation. Soha Ghaffar performed the characterization of materials. Sadaf ul Nisa helped in graphical work and data curation. Sidra Tul Muntaha helped in data interpretation and calculations. Muhammad Danish supervised and designed the project and helped in final editing. Syed Rizwan Shafqat designed the project and investigation. Sumera Arshad helped in evaluating the electrochemical performance and write-up. Sufyan Ashraf contributed to calculations, interpretations and graphical work. Muhammad Asam Raza helped in results and discussion and final reviewing and editing of the write-up.

## Conflicts of interest

All the authors show no conflict of interest.

## Data Availability

The data will be available on reasonable request to the corresponding author.
